# OCTA-based evaluation of the correlation between macular retinal and choroidal structural characteristics and refractive status in patients with retinopathy of prematurity

**DOI:** 10.3389/fped.2025.1642782

**Published:** 2025-11-11

**Authors:** Chen Chen, Liu Hong

**Affiliations:** Ophthalmology Department, Dalian Women and Children’s Medical Group, Dalian, China

**Keywords:** retinopathy of prematurity, optical coherence tomography angiography, macular microcirculation, refractive parameters, retinal structural characteristics, vascular endothelial growth factor

## Abstract

**Purpose:**

To investigate the correlation between macular retinal and choroidal structural characteristics and refractive status in preschool-aged children with retinopathy of prematurity (ROP), based on optical coherence tomography angiography (OCTA), and to analyze the relationship between serum vascular endothelial growth factor (VEGF) levels and microcirculatory alterations.

**Methods:**

310 preterm children with a history of ROP who were followed up at our hospital between January 2022 and June 2024 were included. According to the International Classification of Retinopathy of Prematurity (ICROP), participants were categorized into mild (*n* = 150, stage 1–2 ROP not meeting treatment criteria), moderate (*n* = 150, pre-threshold stage 3 ROP requiring treatment), and severe (*n* = 10, stage 3 threshold or above, or cases with retinal detachment) groups. Foveal avascular zone (FAZ) area, vessel densities of the superficial capillary plexus (SCP) and deep capillary plexus (DCP), choroidal thickness, number of vascular branches, vascular tortuosity, and foveal thickness were evaluated. Refractive parameters and VEGF levels were measured. The correlation between OCTA parameters, refractive parameters, and serum VEGF levels was analyzed.

**Results:**

The myopia rate in the severe group (80.0%) was higher than that in the moderate (52.0%) and mild (18.7%) groups (*χ*² = 68.45, *P* < 0.001). The spherical equivalent (SE) was (−2.35 ± 2.75) D, (−0.85 ± 2.15) D, and (+0.75 ± 1.25) D in the severe, moderate, and mild groups, respectively (*F* = 45.32, *P* < 0.001). The FAZ area was negatively correlated with the SE (*r* = −0.659, *P* < 0.001) and positively correlated with the axial length (*r* = 0.653, *P* < 0.001). The SCP vessel density was positively correlated with the SE (*r* = 0.618, *P* < 0.001). The choroidal thickness was positively correlated with the SE (*r* = 0.655, *P* < 0.001). Multivariable logistic regression analysis showed that a history of severe ROP (OR = 3.85, 95% CI: 2.52–5.86), SCP vessel density <40% (OR = 2.58, 95% CI: 1.78–3.76), FAZ area >0.35 mm² (OR = 2.24, 95% CI: 1.55–3.25), and choroidal thickness <250 μm (OR = 2.32, 95% CI: 1.62–3.36) were associated with the development of myopia. Serum VEGF level was positively correlated with FAZ area (*r* = 0.375, *P* < 0.001) and negatively correlated with SCP vessel density (*r* = −0.419, *P* < 0.001).

**Conclusion:**

Increased FAZ area, decreased SCP vessel density, and decreased choroidal thickness were associated with the development of myopia. Serum VEGF levels were correlated with macular microcirculatory parameters.

## Introduction

Retinopathy of Prematurity (ROP) is a proliferative disorder that disrupts the normal vascular development of the retina in preterm infants and represents one of the leading causes of childhood visual impairment and blindness worldwide (1). With advancements in neonatal intensive care, the survival rates of extremely preterm and very low birth weight infants have markedly improved, leading to a global increase in the incidence of ROP (2). In China, ROP has emerged as a major cause of childhood blindness, bringing a heavy burden to the affected children, their families, and society (3).

Although interventions such as laser photocoagulation and anti-vascular endothelial growth factor (VEGF) therapy can effectively control the progression of ROP in the acute phase, children with ROP still face many long-term visual complications, among which refractive error is particularly common (4, 5). Studies have demonstrated that the long-term incidence of refractive problems in children with ROP is significantly higher than that in full-term children, among which myopia, astigmatism, and hyperopia are the most common refractive anomalies (6). Previous research has indicated a positive correlation between ROP severity and the risk of myopia, with the incidence of myopia reaching up to 80% among children who have undergone treatment for severe ROP (7). However, the exact pathological mechanisms underlying refractive anomalies in ROP remain incompletely elucidated and are likely associated with multiple factors such as aberrant ocular growth patterns and structural changes in the retina.

In recent years, optical coherence tomography angiography (OCTA), a non-invasive and high-resolution imaging modality, has enabled the direct visualization of the retinal and choroidal microcirculation, including parameters such as the foveal avascular zone (FAZ) area, capillary plexus vessel density, and choroidal thickness (8). The application of OCTA provides a novel approach for in-depth investigation of the relationship between retinal microcirculatory alterations and refractive anomalies in children with ROP. Preliminary studies (9) have indicated that parameters such as FAZ area, retinal vessel density, and choroidal thickness in children with ROP differ from those in age-matched children born at term; however, research on the correlation between these microcirculatory structural characteristics and refractive status remains relatively limited. Moreover, as a key factor in the pathogenesis of ROP, whether serum levels of VEGF are associated with late-stage retinal microvascular remodeling and refractive development remains insufficiently investigated.

VEGF plays a pivotal role in the pathogenesis of ROP and is a key factor regulating retinal angiogenesis (10). While the changes in VEGF levels during the acute phase of ROP have been widely studied, there are few studies on serum VEGF levels in the late phase of ROP. Recent studies have found that even years after stabilization of ROP, the serum VEGF levels of some children may still be different from those of normal children (11). A preliminary study on school-age children with a history of ROP found that serum VEGF levels were correlated with retinal vascular density (12). Exploring the relationship between serum VEGF levels, retinal microcirculation, and refractive status in children with a history of ROP has important theoretical and clinical significance.

Based on this, the present study aims to address a core issue: Does a history of ROP affect refractive development in preschool-aged children by changing the macular microcirculatory structure? It should be noted that this study focused on the microcirculatory changes of the retina and choroid in the posterior pole, rather than a comprehensive assessment of changes in all structures of the eye (such as the anterior segment). We chose this research focus based on the following considerations: (1) The macula is the most important area for visual function, and changes in its microcirculation may directly affect visual development; (2) OCTA technology has unique advantages in evaluating microcirculation in the posterior pole; (3) Previous studies have mostly focused on changes in the anterior segment, while few studies have explored the relationship between microcirculation of the posterior pole and refractive development.

## Materials and methods

### Research design and patient selection

This study employed a retrospective cohort design and received approval from the ethics committee of Dalian Women and Children's Medical Group. It adhered to the ethical principles of the Declaration of Helsinki. Data were retrospectively collected from preterm children aged 3–6 years with a history of ROP who had been followed up at our hospital between January 2022 and June 2024. The data were retrieved using the hospital's electronic medical records system based on the following inclusion criteria.

Inclusion criteria: (1) Preterm children aged 3–6 years who met the diagnostic criteria for ROP (13), with a documented history of ROP; (2) Availability of comprehensive ophthalmologic examination data, including OCTA imaging, refractive parameter measurements, and axial length assessment; (3) Complete test results of serum VEGF levels; (4) Thorough medical history, including gestational age, birth weight, underlying diseases, and treatment modalities.

After applying the aforementioned selection criteria, a total of 437 patients were initially included. Subsequently, a secondary screening was performed based on the following exclusion criteria: (1) Presence of other severe ocular diseases (e.g., congenital cataracts, glaucoma, retinal vascular disorders); (2) Comorbidities with systemic diseases that may affect ocular development (e.g., chromosomal abnormalities, metabolic disorders); (3) Patients who did not undergo OCTA or refractive examinations; (4) Incomplete data or loss to follow-up; (5) Recent use (within the past 3 months) of systemic medications that may affect ocular vascular development.

A total of 310 patients were finally included according to the aforementioned criteria. Based on the International Classification of Retinopathy of Prematurity (ICROP) (14), the included children were categorized into three groups: mild (*n* = 150, stages 1–2, not meeting treatment criteria), moderate (*n* = 150, pre-threshold stage 3 requiring treatment), and severe (*n* = 10, threshold stage 3 or higher, or cases with retinal detachment).

## Data collection

### Baseline information

Demographic data of the children (sex, age), birth information (gestational age, birth weight), ROP-related parameters (stage, zone, treatment modality), and follow-up records were collected.

### Ocular examinations

#### OCTA examination and parameter measurement

OCTA was performed using the AngioVue OCTA system or the RTVue XR Avanti device (Optovue Inc., Fremont, CA, USA). To ensure the consistency of the measurement results of the two devices, cross-validation was performed between the devices before the starting of the study. Twenty children were scanned using both devices. Bland-Altman analysis was performed on the main parameters (FAZ area and vessel density). The results showed that the 95% limits of agreement of the measurements between the two devices were within the clinically acceptable range [FAZ area: −0.02 to 0.03 mm²; superficial capillary plexus (SCP) vessel density: −1.5% to 1.8%]. Based on the validation results, we applied device-specific correction factors: FAZ area measured by the RTVue XR Avanti was multiplied by 0.98, and vessel density was multiplied by 1.02 to standardize the values measured by the AngioVue system.

A 3 × 3 mm macular region was scanned. All OCTA parameters were automatically measured using the built-in AngioAnalytics software (version 2017.1.0.151). The software uses the split-spectrum amplitude-decorrelation angiography (SSADA) algorithm to automatically identify blood vessels and calculate vessel density. The FAZ area was measured using the automatic identification function of the software, and the vessel density was defined as the percentage of vessel pixels to the total pixels in the selected area. For images with obvious errors in the automatic measurement results (e.g., incorrect FAZ boundary detection), two experienced technicians corrected them using the manual adjustment function of the software.

#### Image segmentation verification and adjustment

Considering the limited cooperation of children and the potential retinal structural abnormalities caused by ROP, the following strategies were adopted to ensure segmentation accuracy: First, all scans were performed by experienced technicians, with each eye scanned at least three times, and images with a signal intensity index ≥7/10 and no obvious motion artifacts were selected for analysis. Second, the automatic layer segmentation function of AngioAnalytics software was used, which identified four retinal layers: the SCP (from 3 μm below the internal limiting membrane to 15 μm below the inner plexiform layer) and the deep capillary plexus (DCP) (from 15 μm below the inner plexiform layer to 70 μm below the inner plexiform layer). Third, all segmentation results were manually reviewed, with particular attention paid to the segmentation accuracy of the foveal center. Fourth, for images with evident segmentation errors (estimated at approximately 15%), two senior ophthalmologists manually corrected them using the software's layer adjustment tool until each segmentation line accurately aligned with the corresponding anatomical interface. Fifth, the adjusted images were then independently reviewed and confirmed by a third ophthalmologist.

#### Correction for refractive parameters

Based on previous studies, magnification correction was applied to OCTA measurements. The Littmann and Bennett formulas were used to correct the magnification effects of different axial lengths and refractive states: Corrected area = Measured area × (Axial length/23.95)²; Corrected vessel density = Measured density × (23.95/Axial length). In this study, approximately 35% of the children required this correction.

### Measurement of serum VEGF levels

To evaluate the persistent changes in VEGF levels in the later stages of ROP, this study retrospectively analyzed the serum VEGF data of the children during the follow-up period. According to our hospital's ROP follow-up protocol, all children with moderate to severe ROP underwent serum VEGF testing annually between the ages of 3 and 6 years as part of long-term monitoring. For the 310 children included in this study, we obtained serum VEGF data within 3 months before and after the most recent OCTA examination. Among them, 68 children had two or more VEGF measurements. We conducted a subgroup analysis of these children and found that the annual coefficient of variation of their VEGF levels was less than 15%, suggesting relative stability of VEGF levels in this age group. Therefore, it was reasonable to use single time point measurements for analysis. All serum samples were collected in the early morning in the fasting state and analyzed using ELISA, with a detection range of 31.2–2,000 pg/ml, an intra-assay coefficient of variation below 5%, and an inter-assay coefficient of variation below 8%.

### Outcome evaluation

The primary outcome measures include: (1) the correlation between macular OCTA parameters (FAZ area, SCP vessel density, and choroidal thickness) and refractive parameters (spherical equivalent and axial length); (2) the differences in OCTA parameters among groups with different degrees of myopia.

The secondary outcome measures include: (1) the distribution of refractive status in different ROP severity groups; (2) the correlation between serum VEGF levels and OCTA parameters; (3) the analysis of risk factors for myopia.

### Statistical methods

All data were analyzed using SPSS version 25.0. Continuous variables were expressed as mean ± standard deviation, and intergroup comparisons were performed using one-way ANOVA or independent sample *t*-tests. Categorical variables were presented as number of cases (percentages), and intergroup comparisons were conducted using the chi-square test or Fisher's exact test. Pearson correlation analysis was employed to evaluate the correlation between OCTA parameters, refractive parameters, and serum VEGF levels. Multivariable logistic regression analysis was conducted to identify independent risk factors for myopia, with odds ratios (ORs) and 95% confidence intervals (CIs) calculated. To distinguish the independent effects of ROP severity and refractive status, analysis of covariance (ANCOVA) was used to control for refractive parameters, and subgroup analysis stratified by refractive status was performed. *P* < 0.05 was considered statistically significant. For multiple comparisons, the Bonferroni correction was applied to adjust *P*-values. In the sensitivity analysis, OCTA parameters were also analyzed as continuous variables, and the results were consistent with those of the categorical variable analysis.

The sample size calculation was based on previous research findings, with *α* = 0.05 and *β* = 0.10. The minimum required sample size for each group was determined based on the differences in the primary outcome measures and standard deviations across the study groups. As a result, the final sample sizes were set at 150 for the mild group, 150 for the moderate group, and 10 for the severe group.

## Results

### Comparison of baseline data

This study included a total of 310 preterm children with a history of ROP, who were categorized into three groups based on the severity of ROP: mild (*n* = 150), moderate (*n* = 150), and severe (*n* = 10). There were no statistically significant differences between the groups in terms of sex and average age (*P* > 0.05), indicating good comparability. However, the severe group had lower gestational age and birth weight compared to the other two groups (*P* < 0.05), suggesting that the extent of prematurity may be associated with the severity of ROP. Additionally, 90.0% of children in the severe group underwent laser therapy, and 80.0% received anti-VEGF treatment, both significantly higher than in the mild and moderate groups (*P* < 0.05), as shown in Table 1. These differences suggest a potential correlation between the severity of ROP, the extent of prematurity, and the need for therapeutic interventions.

**Table 1:** Comparison of basic characteristics of study participants (mean ± SD)/[*n* (%)].

General clinical data	Mild group (*n* = 150)	Moderate group (*n* = 150)	Severe group (*n* = 10)	*P*
Average age (years)	4.21 ± 0.82	4.35 ± 0.92	4.11 ± 0.72	0.156
Sex (male/female)	78/72	80/70	6/4	0.713
Gestational age (weeks)	31.21 ± 1.82	29.32 ± 1.53	26.71 ± 1.35	<0.001
Birth weight (g)	1,580 ± 320	1,320 ± 280	980 ± 210	<0.001
Laser therapy (%)	0 (0%)	68 (45.3%)	9 (90.0%)	<0.001
Anti-VEGF treatment (%)	0 (0%)	32 (21.3%)	8 (80.0%)	<0.001

### Comparison of refractive parameters among groups with varying severity of ROP

The assessment of refractive status revealed significant differences in refractive parameters among the three groups of children with ROP. The spherical equivalent in the severe group (−2.35  ± 2.75 D) was markedly lower than that in the moderate (−0.85 ± 2.15 D) and mild groups (+0.75 ± 1.25 D) (*P* < 0.001), indicating a greater predisposition toward myopia in children with severe ROP. Astigmatism (−1.85 ± 0.95 D) and axial length (23.15 ± 0.95 mm) were also significantly greater in the severe group compared to the other two groups (*P* < 0.001). The incidence of myopia in the severe group reached 80.0%, significantly exceeding that of the moderate (52.0%) and mild groups (18.7%) (*P* < 0.001). In contrast, the incidence of hyperopia exhibited an opposite trend, with only 10.0% in the severe group, significantly lower than 56.7% observed in the mild group (*P* < 0.001). The incidence of astigmatism (>1.0 D) in the severe group (80.0%) was also significantly higher than in the moderate (59.3%) and mild groups (28.0%) (*P* < 0.001), as shown in Table 2. These findings suggest a potential correlation between the severity of ROP and the incidence of myopia, astigmatism, and axial elongation.

**Table 2:** Comparison of refractive parameters among groups with varying severity of ROP.

Refractive parameter	Mild group (*n* = 150)	Moderate group (*n* = 150)	Severe group (*n* = 10)	*P*
Spherical equivalent (D)	+0.75 ± 1.25	−0.85 ± 2.15	−2.35 ± 2.75	<0.001
Cylindrical power (D)	−0.65 ± 0.45	−1.15 ± 0.65	−1.85 ± 0.95	<0.001
Axial length (mm)	21.85 ± 0.65	22.45 ± 0.75	23.15 ± 0.95	<0.001
Proportion of myopia (%)	28 (18.7%)	78 (52.0%)	8 (80.0%)	<0.001
Proportion of hyperopia (%)	85 (56.7%)	42 (28.0%)	1 (10.0%)	<0.001
Proportion of astigmatism (>1.0 D, %)	42 (28.0%)	89 (59.3%)	8 (80.0%)	<0.001

### Macular retinal and choroidal structural characteristics assessed by OCTA

OCTA findings revealed significant differences in the macular microcirculation structure among the three groups of ROP patients. The FAZ area in the severe group (0.42 ± 0.15 mm²) was significantly larger than that in the moderate (0.32 ± 0.12 mm²) and mild groups (0.25 ± 0.08 mm²) (*P* < 0.001). The vessel densities of the SCP (38.6 ± 4.8)% and DCP (46.5 ± 5.1)% were markedly lower in the severe group compared to the moderate and mild groups (*P* < 0.001, *P* = 0.005, respectively). Furthermore, choroidal thickness (245.8 ± 45.2 μm) and retinal nerve fiber layer thickness (98.3 ± 12.5 μm) were also significantly thinner in the severe group compared to the moderate and mild groups (*P* < 0.001, *P* = 0.003). No statistically significant difference was observed in foveal thickness among the three groups (*P* = 0.113) (Table 3). These results indicate that children with severe ROP exhibit more pronounced structural abnormalities in the macular microcirculation, primarily characterized by FAZ enlargement, microvascular network rarefaction, and thinning of the choroid and nerve fiber layer.

**Table 3:** Macular retinal and choroidal structural characteristics assessed by OCTA.

Refractive parameter	Mild group (*n* = 150)	Moderate group (*n* = 150)	Severe group (*n* = 10)	*P*
FAZ area (mm²)	0.25 ± 0.08	0.32 ± 0.12	0.42 ± 0.15	<0.001
SCP vessel density (%)	48.51 ± 3.22	43.21 ± 4.12	38.61 ± 4.82	<0.001
DCP vessel density (%)	52.31 ± 3.53	49.85 ± 4.21	46.51 ± 5.12	0.005
Choroidal thickness (μm)	325.51 ± 38.62	285.21 ± 42.52	245.81 ± 45.23	<0.001
Foveal thickness (μm)	238.52 ± 21.63	235.32 ± 25.41	232.61 ± 28.32	0.113
Retinal nerve fiber layer thickness (μm)	110.21 ± 8.52	105.62 ± 9.33	98.35 ± 12.59	0.003

### Correlation analysis between macular structural characteristics and refractive parameters

**Figure 1:**
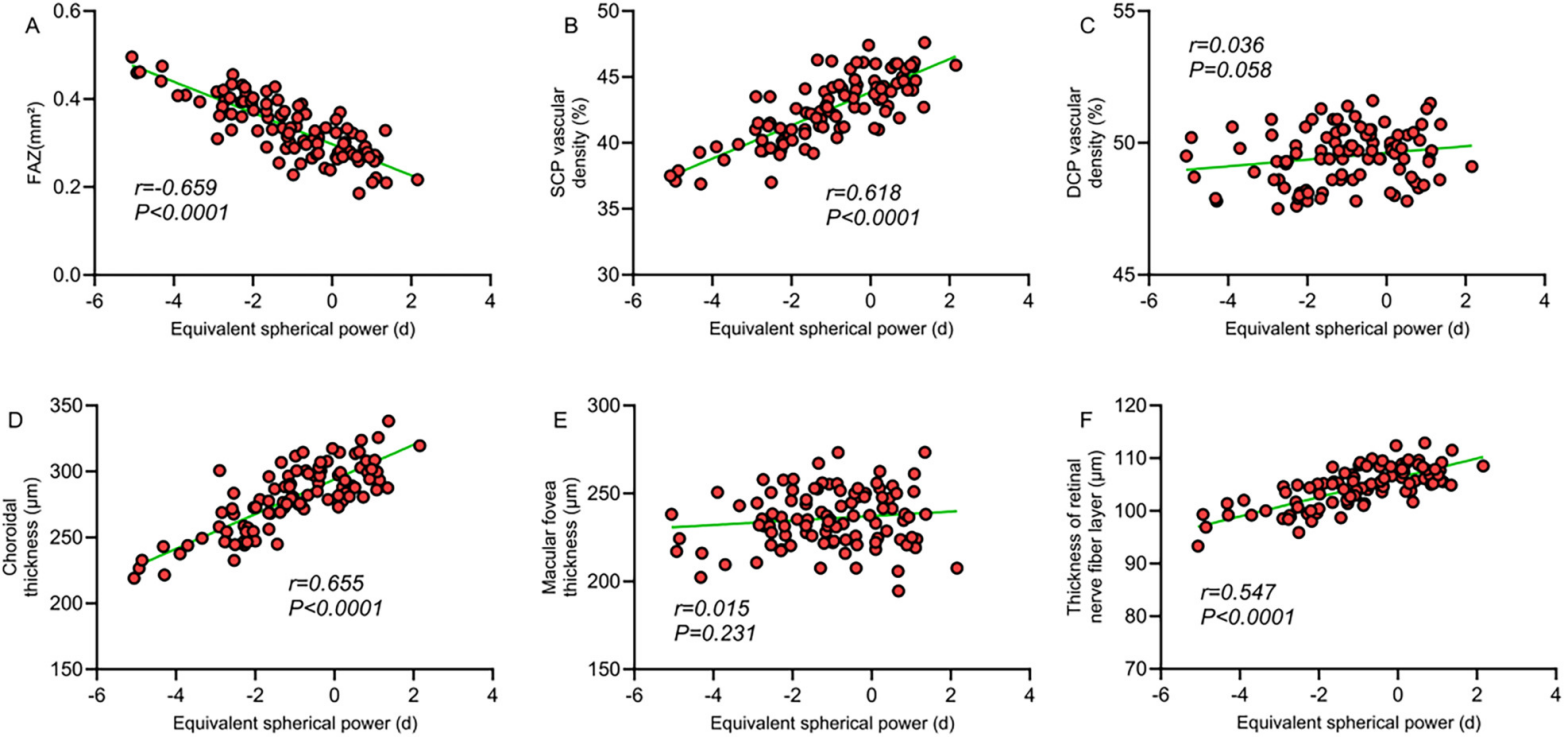
**(A)** Correlation between FAZ area and spherical equivalent; **(B)** correlation between SCP vessel density and spherical equivalent; **(C)** correlation between DCP vessel density and spherical equivalent; **(D)** correlation between choroidal thickness and spherical equivalent; **(E)** correlation between macular foveal thickness and spherical equivalent; **(F)** correlation between retinal nerve fiber layer thickness and spherical equivalent.

**Figure 2:**
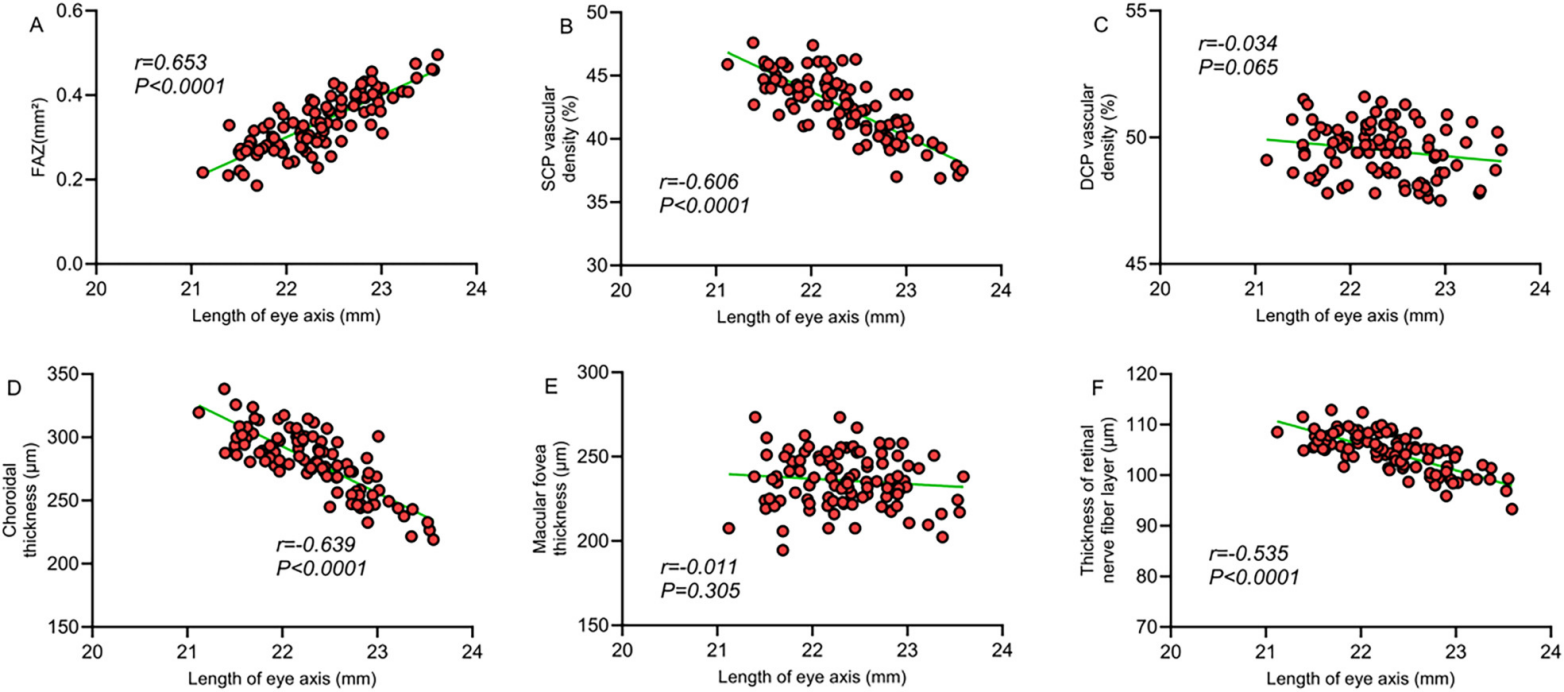
**(A)** Correlation between FAZ area and axial length; **(B)** correlation between SCP vessel density and axial length; **(C)** correlation between DCP vessel density and axial length; **(D)** correlation between choroidal thickness and axial length; **(E)** correlation between macular foveal thickness and axial length; **(F)** correlation between retinal nerve fiber layer thickness and axial length.

Correlation analysis revealed a significant correlation between macular structural characteristics and refractive parameters in children with ROP. The FAZ area was significantly negatively correlated with spherical equivalent (*r* = −0.659, *P* < 0.001) and positively correlated with axial length (*r* = 0.653, *P* < 0.001), indicating that a larger FAZ area is associated with higher myopic severity and increased axial length. SCP vessel density showed a significant positive correlation with spherical equivalent (*r* = 0.618, *P* < 0.001) and a significant negative correlation with axial length (*r* = −0.606, *P* < 0.001), suggesting that decreased SCP vessel density is linked to myopia progression and axial elongation. Similarly, choroidal thickness was significantly positively correlated with spherical equivalent (*r* = 0.655, *P* < 0.001) and negatively correlated with axial length (*r* = −0.639, *P* < 0.001). However, no significant correlations were observed between DCP vessel density or foveal thickness and refractive parameters (*P* > 0.05). A moderate correlation was also found between retinal nerve fiber layer thickness and refractive status (*P* < 0.05). These findings suggest that alterations in macular microcirculation may be closely related to abnormal refractive development in children with ROP (Table 4; Figures 1, 2).

**Table 4:** Correlation analysis between macular structural characteristics and refractive parameters.

OCTA parameter	Spherical equivalent		Axial length	
	*r*	*P*	*r*	*P*
FAZ area	−0.659	<0.0001	0.653	<0.0001
SCP vessel density	0.618	<0.0001	−0.606	<0.0001
DCP vessel density	0.036	0.058	−0.034	0.065
Choroidal thickness	0.655	<0.0001	−0.639	<0.0001
Foveal thickness	0.015	0.231	−0.011	0.305
Retinal nerve fiber layer thickness	0.547	<0.0001	−0.535	<0.0001

The r value represents the Pearson correlation coefficient; a positive value indicates a positive correlation, while a negative value indicates a negative correlation.

### Comparison of macular microcirculatory characteristics among groups with different degrees of myopia

Analysis stratified by myopia severity revealed that the FAZ area in the moderate-to-high myopia group (*n* = 31) was significantly larger (0.45 ± 0.16 mm²) compared to the mild myopia group (0.35 ± 0.12 mm²) and the non-myopic group (0.26 ± 0.09 mm²) (*P* < 0.001). The SCP vessel density was markedly lower in the moderate-to-high myopia group (37.8 ± 4.5%) than in the mild myopia (42.5 ± 3.9%) and non-myopia groups (48.2 ± 3.5%) (*P* < 0.001). Choroidal thickness was also significantly thinner in the moderate-to-high myopia group (235.5 ± 42.8 μm) compared to the other two groups (*P* < 0.001). Moreover, the number of vascular branches (28.6 ± 5.2 branches/mm²) was significantly lower, while vascular tortuosity (1.25 ± 0.13) was markedly higher in the moderate-to-high myopia group compared to the other two groups (*P* < 0.001 and *P* = 0.003, respectively) (Table 5). These findings indicate a strong positive correlation between the degree of myopia and the extent of macular microcirculatory abnormalities in children with ROP; children with more severe myopia exhibit more pronounced microcirculatory structural changes.

**Table 5:** Comparison of macular microcirculatory characteristics among groups with different degrees of myopia.

OCTA parameter	Non-myopia group (*n* = 201)	Mild myopia group (*n* = 78)	Moderate-to-high myopia group (*n* = 31)	*P*
FAZ area (mm²)	0.26 ± 0.09	0.35 ± 0.12	0.45 ± 0.16	<0.001
SCP vessel density (%)	48.2 ± 3.5	42.5 ± 3.9	37.8 ± 4.5	<0.001
DCP vessel density (%)	51.5 ± 3.8	49.2 ± 4.3	47.3 ± 4.9	0.059
Choroidal thickness (μm)	322.6 ± 36.5	278.3 ± 40.2	235.5 ± 42.8	<0.001
Number of vascular branches (branches/mm²)	36.5 ± 4.2	32.8 ± 4.5	28.6 ± 5.2	<0.001
Vascular tortuosity	1.12 ± 0.08	1.18 ± 0.10	1.25 ± 0.13	0.003

The non-myopia group is defined as SE ≥ 0.50 D; the mild myopia group as −0.50 D ≥ SE ≥ 3.00 D; and the moderate-to-high myopia group as SE ≤ −3.00 D.

### Multivariable logistic regression analysis of risk factors for myopia in children with ROP

Multivariable logistic regression analysis identified the following independent risk factors for the development of myopia in children with ROP: a history of severe ROP (OR = 3.85, 95% CI: 2.52–5.86, *P* < 0.001), gestational age <28 weeks (OR = 2.65, 95% CI: 1.85–3.78, *P* < 0.001), birth weight <1,000 g (OR = 2.32, 95% CI: 1.62–3.41, *P* < 0.001), reduced SCP vessel density (<40%) (OR = 2.58, 95% CI: 1.78–3.76, *P* < 0.001), increased FAZ area (>0.35 mm²) (OR = 2.24, 95% CI: 1.55–3.25, *P* < 0.001), decreased choroidal thickness (<250 μm) (OR = 2.32, 95% CI: 1.62–3.36, *P* < 0.001), history of laser therapy (OR = 2.15, 95% CI: 1.48–3.12, *P* = 0.002), and anti-VEGF treatment (OR = 1.85, 95% CI: 1.26–2.73, *P* = 0.008) (Table 6). These findings suggest that the severity of ROP, extent of prematurity, alterations in macular microvascular structure, and therapeutic interventions are all critical factors influencing the development of myopia in children with ROP, with a history of severe ROP posing the highest risk.

**Table 6:** Multivariable logistic regression analysis of risk factors for myopia in children with ROP.

Factor	*B*	S.E	Wald	*P*	OR	95% CI
Gestational age <28 weeks	0.975	0.183	28.369	<0.001	2.65	1.85–3.78
Birth weight <1,000 g	0.842	0.198	18.054	<0.001	2.32	1.62–3.41
History of severe ROP	1.348	0.214	39.715	<0.001	3.85	2.52–5.86
History of laser therapy	0.766	0.190	16.243	0.002	2.15	1.48–3.12
History of anti-VEGF treatment	0.615	0.199	9.567	0.008	1.85	1.26–2.73
Increased FAZ area (>0.35 mm²)	0.806	0.191	17.828	<0.001	2.24	1.55–3.25
Reduced SCP vessel density (<40%)	0.948	0.193	24.115	<0.001	2.58	1.78–3.76
Decreased choroidal thickness (<250 μm)	0.842	0.190	19.657	<0.001	2.32	1.62–3.36

OR denotes the odds ratio; CI represents the confidence interval.

### Correlation analysis between serum VEGF levels and OCTA parameters

**Figure 3:**
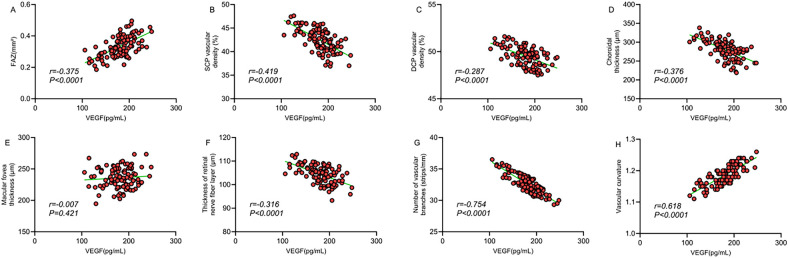
**(A)** Correlation between FAZ area and VEGF; **(B)** correlation between SCP vessel density and VEGF; **(C)** correlation between DCP vessel density and VEGF; **(D)** correlation between choroidal thickness and VEGF; **(E)** correlation between macular foveal thickness and VEGF; **(F)** correlation between retinal nerve fiber layer thickness and VEGF; **(G)** correlation between number of vascular branches and VEGF; **(H)** correlation between vascular tortuosity and VEGF.

The correlation analysis revealed that serum VEGF levels exhibited a significant positive correlation with FAZ area (*r* = 0.375, *P* < 0.0001), a significant negative correlation with SCP vessel density (*r* = −0.419, *P* < 0.0001), a mild negative correlation with DCP vessel density (*r* = −0.287, *P* = 0.042), and a moderate negative correlation with choroidal thickness (*r* = −0.376, *P* < 0.0001). Moreover, serum VEGF levels were significantly negatively correlated with the number of vascular branches (*r* = −0.754, *P* < 0.0001), moderately positively correlated with vascular tortuosity (*r* = 0.618, *P* < 0.0001), and moderately negatively correlated with retinal nerve fiber layer thickness (*r* = −0.315, *P* = 0.008), but showed no significant correlation with foveal thickness (*r* = 0.007, *P* = 0.421) (Table 7; Figure 3). These findings suggest that serum VEGF levels may involve in regulating retinal microcirculatory development in children with ROP, with elevated VEGF levels closely associated with structural abnormalities in the macular microcirculation.

**Table 7:** Correlation analysis between serum VEGF levels and OCTA parameters.

OCTA parameters	VEGF level	
	*r*	*P*
FAZ area	0.375	<0.0001
SCP vessel density	−0.419	<0.0001
DCP vessel density	−0.287	<0.0001
Choroidal thickness	−0.376	<0.0001
Foveal thickness	0.007	0.421
Retinal nerve fiber layer thickness	−0.316	<0.0001
Number of vascular branches	−0.754	<0.0001
Vascular tortuosity	0.618	<0.0001

### Comparison of retinal structural characteristics in children with ROP under different treatment modalities

ROP patients were stratified into subgroups based on treatment modalities. Comparative analysis revealed significant differences in OCTA parameters among groups. The FAZ area in the combination therapy group (laser + anti-VEGF, *n* = 33) was significantly larger [(0.42 ± 0.16) mm²] than that in the laser-only group [(0.35 ± 0.14) mm²], anti-VEGF-only group [(0.32 ± 0.12) mm²], and untreated group [(0.25 ± 0.09) mm²] (*P* < 0.001). The SCP vessel density in the combination therapy group [(36.8 ± 5.1)%] was significantly lower compared to the other three groups (*P* < 0.001), and the DCP vessel density [(44.2 ± 5.2)%] was markedly lower than that of the untreated and anti-VEGF-only groups (*P* = 0.003). Additionally, choroidal thickness in the combination therapy group [(238.5 ± 48.2) μm] was significantly reduced compared to the other three groups (*P* < 0.001). Regarding refractive status, the spherical equivalent in the combination therapy group [(−2.65 ± 2.85) D] was significantly lower compared to the other three groups (*P* < 0.001), indicating a higher degree of myopia in this subgroup. No statistically significant difference was observed among the four groups in terms of foveal thickness (*P* = 0.112) (Table 8). These findings suggest that ROP children receiving more aggressive treatment may exhibit more pronounced structural abnormalities in the macular microcirculation and more severe myopia, which may be attributed to the combined effects of underlying disease severity and therapeutic interventions.

**Table 8:** Comparison of retinal structural characteristics in children with ROP under different treatment modalities.

OCTA parameter	Untreated group (*n* = 220)	Laser-only group (*n* = 95)	Anti-VEGF-only group (*n* = 52)	Combination therapy group (*n* = 33)	*P*
FAZ area (mm²)	0.25 ± 0.09	0.35 ± 0.14	0.32 ± 0.12	0.42 ± 0.16	<0.001
SCP vessel density (%)	47.82 ± 3.63	39.55 ± 4.45	42.35 ± 4.25	36.84 ± 5.15	<0.001
DCP vessel density (%)	51.51 ± 3.92	46.22 ± 4.58	48.56 ± 4.52	44.25 ± 5.25	0.003
Choroidal thickness (μm)	315.65 ± 42.55	255.35 ± 45.84	275.36 ± 43.65	238.56 ± 48.25	<0.001
Foveal thickness (μm)	238.25 ± 22.54	232.54 ± 25.85	235.65 ± 24.24	228.35 ± 27.52	0.112
Spherical equivalent (D)	+0.65 ± 1.45	−1.85 ± 2.35	−1.25 ± 2.15	−2.65 ± 2.85	<0.001

### Relationship between ROP severity and OCTA parameters after controlling for refractive factors

To distinguish the independent effects of ROP severity and refractive status on OCTA parameters, refractive parameters (spherical equivalent and axial length) were used as covariates to analyze the differences in OCTA parameters between ROP severity groups. The results showed that after controlling for refractive factors, ROP severity was significantly correlated with FAZ area (*F* = 15.32, *P* < 0.001), SCP vessel density (*F* = 12.85, *P* < 0.001), and choroidal thickness (*F* = 18.79, *P* < 0.001). These findings suggest that the effect of ROP severity on microcirculatory outcomes is independent of refractive status.

### Subgroup analysis

The children were stratified into emmetropic subgroup (SE ≥ 0.50 D) and myopia subgroup (SE ≤ −0.50 D) according to refractive status. OCTA parameters of ROP severity were compared within the subgroups. The results showed that in the emmetropic subgroup (*n* = 21), the FAZ area of children with severe ROP [(0.35 ± 0.12) mm^2^] was still larger than that of children with mild ROP [(0.24 ± 0.08) mm^2^] (*P* < 0.05). In the myopia subgroup (*n* = 179), the SCP vessel density in children with severe ROP [(36.51 ± 4.21) %] was lower than that in children with mild ROP [(44.81 ± 3.81) %] (*P* < 0.05).

## Discussion

This study, utilizing OCTA technology, systematically investigated for the first time the correlation between macular retinal and choroidal structural characteristics and refractive status in children with a history of ROP. It also examined the association between serum VEGF levels and microcirculatory changes. The results indicated a strong correlation between the severity of ROP and the risk of developing myopia, with a significantly higher incidence of myopia in children with severe ROP compared to those with moderate or mild ROP. The microcirculatory structural characteristics in the macular region, particularly FAZ enlargement, reduced vessel density in the SCP, and decreased choroidal thickness, were significantly associated with the onset and progression of myopia. Furthermore, serum VEGF levels may influence the development of retinal and choroidal microcirculation, thereby contributing to the regulation of refractive status in children with ROP. These findings provide novel insights into the structural basis of refractive anomalies in children with ROP and may serve as a foundation for early prediction and intervention strategies targeting long-term refractive development.

### Severity of ROP and risk of myopia

This study revealed that the incidence of myopia reached 80.0% in the severe group, significantly higher than that in the moderate (52.0%) and mild (18.7%) groups. A history of severe ROP emerged as the most potent independent risk factor for the development of myopia (OR = 3.85), consistent with previous findings. Wang et al. (15), through a refractive status analysis of preterm children aged 7 years, demonstrated a strong association between prematurity, low birth weight, and refractive status compared to children born at term. Moreover, significant differences in corneal power, corneal curvature, and axial length were found between children with ROP and those in other groups. Similarly, Xie et al. (16) categorized preterm children into ROP and non-ROP groups and found that preterm birth increased the risk of myopia compared to full-term birth, with ROP children being more susceptible to the concurrent development of myopia and astigmatism. We propose that the observed correlation may stem from several underlying factors. First, ROP is inherently a disorder characterized by aberrant neovascularization, and the severity of the disease exacerbates disruption to the retinal microcirculation, thereby impairing normal refractive development. Second, severe ROP is more prevalent among infants born at earlier gestational ages with lower birth weights, and the extent of prematurity is closely associated with axial length growth and refractive developmental anomalies. Third, children with advanced ROP often require interventions such as laser photocoagulation or anti-VEGF therapy, which may exert additional effects on ocular development.

Compared with previous studies, the uniqueness of this research lies in our more detailed quantification of refractive parameters in children with varying severities of ROP, including spherical equivalent, cylindrical power, and axial length. We found that children with severe ROP not only had a higher incidence of myopia, but also exhibited greater myopic severity, higher astigmatism, and longer axial lengths. These quantitative data provide a more precise depiction of the impact of ROP on refractive development, thereby enabling clinicians to more accurately assess the refractive risk in affected children.

### Correlation between macular microcirculation structure and refractive status

This study, using OCTA technology, systematically evaluated for the first time the correlation between macular microcirculation structure and refractive status in children with ROP, identifying FAZ area, SCP vessel density, and choroidal thickness as key indicators closely associated with refractive outcomes.

### FAZ area and refractive status

The study results indicated that FAZ area was significantly negatively correlated with spherical equivalent (*r* = −0.659) and significantly positively correlated with axial length (*r* = 0.653), suggesting that a larger FAZ area is associated with higher myopic severity and increased axial length. We propose that this may be attributed to early hypoxia and abnormal angiogenesis, leading to impaired vascular development in the macular region, which subsequently affects FAZ formation and ocular refractive development. Moreover, the study found significant differences in FAZ area among ROP patients with different degrees of myopia, with the moderate-to-high myopia group exhibiting markedly larger FAZ areas compared to the mild myopia and non-myopic groups. This gradient further corroborates the close association between FAZ area and myopia progression. Although previous studies (17) have reported similar findings, their subjects were primarily adults. This study is the first to confirm the association in preschool-aged children, suggesting that FAZ enlargement may represent an early microcirculatory alteration linked to myopia development in ROP patients, and may serve as a potential predictive biomarker for myopia progression.

### SCP vessel density and refractive status

This study revealed that SCP vessel density was significantly positively correlated with spherical equivalent (*r* = 0.618) and significantly negatively correlated with axial length (*r* = −0.606), indicating that reduced SCP vessel density is associated with higher degrees of myopia and increased axial elongation. Multivariable analysis identified an SCP vessel density of less than 40% as an independent risk factor for the development of myopia (OR = 2.58). This finding aligns with previous studies (18, 19), which reported reduced retinal vascular density in myopic patients compared to normal controls. However, these earlier investigations primarily focused on pathological myopia in adults or older children. In contrast, this study is the first to demonstrate such an association in preschool-aged children with a history of ROP, shedding light on a potential mechanism underlying the development of myopia following ROP.

Furthermore, this study also revealed a significant correlation between SCP vessel density and refractive status, whereas no significant correlation was found between DCP vessel density and refractive parameters. This selective correlation may be attributed to the distinct roles of the SCP and DCP in retinal development and function. The SCP is primarily responsible for supplying blood to the retinal nerve fiber layer and ganglion cell layer, both of which play critical roles in visual function and ocular development. Therefore, structural changes in these regions may directly influence refractive development.

### Choroidal thickness and refractive status

This study found a significant positive correlation between choroidal thickness and spherical equivalent (*r* = 0.655), and a significant negative correlation with axial length (*r* = −0.639). Choroidal thickness <250 μm was identified as an independent risk factor for myopia development (OR = 2.32). Previous research (20) has reported that choroidal thickness is reduced in myopic individuals, suggesting that this may be one of the mechanisms underlying axial elongation in myopia. Changes in the thickness of choroid, an intermediate layer of the ocular wall, may reflect alterations in the biomechanical properties of the ocular wall and affect axial growth.

This study is the first to systematically evaluate the association between choroidal thickness and refractive status in children with ROP. We found that the choroidal thickness in the severe group (245.8 ± 45.2 μm) was significantly lower than in the moderate (285.2 ± 42.5 μm) and mild (325.5 ± 38.6 μm) groups. This gradation suggests that ROP may contribute to the onset of refractive anomalies by influencing choroidal development. The choroid is the most highly vascularized structure in the eye, and its development is closely associated with angiogenic factors such as VEGF. Given that the pathogenesis of ROP involves aberrant expression of these factors, it is possible that ROP indirectly affects refractive status by disrupting choroidal development.

In addition, the study further verified the independent effect of ROP severity on OCTA parameters through ANCOVA and subgroup analysis. Even after controlling for refractive parameters, significant differences in microcirculation were observed among groups with varying severity of ROP. These results suggest that the observed microcirculatory changes are not merely secondary changes of refractive error, but may reflect the persistent impact of ROP on retinal development. This supports the hypothesis that ROP may influence refractive development by directly affecting the retinal microcirculatory structure.

### Association between serum VEGF levels and retinal microcirculation changes

This study is the first to systematically analyze the correlation between serum VEGF levels and retinal microcirculatory structural characteristics in children with ROP. Serum VEGF levels were significantly positively correlated with FAZ area (*r* = 0.375), significantly negatively correlated with SCP vessel density (*r* = −0.419) and number of vascular branches (*r* = −0.754), and moderately positively correlated with vascular tortuosity (*r* = 0.618). These findings suggest that serum VEGF levels may play a role in the regulation and remodeling of retinal microcirculation development in children with ROP. We posit that VEGF, as a pivotal angiogenic factor, plays a central role in the pathogenesis of ROP. Previous studies (21) have demonstrated the bidirectional regulatory effects of dynamic VEGF fluctuations on retinal vascular development during ROP progression. Building upon this theoretical framework, the present study further reveals a significant association between serum VEGF levels and the structural characteristics of macular microcirculation, indicating that VEGF may exert a sustained influence on retinal microcirculatory remodeling in the later stages of ROP.

Compared with previous studies, the innovation of this research lies in the direct correlation analysis between serum VEGF levels and OCTA parameters, revealing a potential association among VEGF levels, macular microcirculatory abnormalities, and the risk of myopia. We conclude that serum VEGF may serve as a potential biomarker connecting ROP with long-term refractive anomalies.

### Correlation of treatment modalities with retinal structure and refractive status

This study compared the retinal structure and refractive status of children with ROP who underwent different treatment modalities, revealing that those who received the combination therapy (laser + anti-VEGF) exhibited the most pronounced changes in macular microcirculation and the highest degree of myopia. While this finding partially aligns with previous clinical observations, some discrepancies remain. Hwang et al. (22), in a retrospective analysis of 78 preterm infants with ROP, investigated the correlation between the number of laser treatment and myopia progression, and their results indicated that eyes receiving more laser spots experienced significantly greater refractive changes, with an average increase of 0.30 diopters of myopia per 1,000 spots annually. The discrepancies between this study and prior research may be attributed to several factors: (1) infants undergoing combination therapy typically present with more severe disease, and thus, the underlying pathology may exert a greater influence on refractive development; (2) insufficient response to anti-VEGF therapy may necessitate additional laser treatment, leading to a more substantial cumulative therapeutic impact; (3) laser and anti-VEGF therapies affect retinal and choroidal vasculature through distinct mechanisms, and their combination may yield a synergistic effect, further influencing refractive development.

Furthermore, the regression analysis in this study indicated that a history of laser therapy (OR = 2.15) and anti-VEGF treatment (OR = 1.85) were both independent risk factors for the development of myopia, suggesting that the interventions may exert an influence on refractive development. This finding aligns with the study by Asano et al. (23), which showed that peripheral retinal damage from laser therapy and the potential disruption of normal vascular development by anti-VEGF treatment may have long-term effects on ocular growth. Therefore, the long-term impact on refractive development should be incorporated as a factor in the decision-making process for ROP treatment.

### Research innovation, limitations, and future directions

This study presents several key innovations. First, it systematically evaluates the correlation between the macular retinal and choroidal structural characteristics and refractive status in preschool-aged children with a history of ROP, providing novel insights into the structural basis of long-term refractive anomalies in ROP children. Second, it analyzes the correlation between serum VEGF levels and retinal microcirculation changes in ROP children, uncovering the potential role of VEGF in the remodeling of retinal microcirculation and regulation of refractive development in the later stages of ROP. Third, through OCTA technology, it quantitatively assesses the correlation between multiple microcirculatory parameters and refractive status, identifying FAZ area, SCP vascular density, and choroidal thickness as potential biomarkers for myopia prediction.

Several limitations of this study should be acknowledged. First, the retrospective design limits causal inferences, preventing the determination of whether the observed retinal microcirculatory changes are a cause or a consequence of myopia. Second, the serum VEGF levels represent only a single time point and do not capture the dynamic fluctuations of VEGF throughout the progression of ROP, potentially affecting the accuracy of the correlation analysis. Third, the study participants were preschool-aged children, whose refractive status was still in the developmental stage, making long-term refractive outcomes uncertain. Fourth, as a single-center study with a relatively small sample size, the statistical power for some subgroup analyses may be affected. Fifth, OCTA technology has inherent limitations, including a restricted scanning range and susceptibility to artifacts, which may compromise the precise assessment of microcirculatory parameters. Finally, this study only evaluated the posterior pole retinal and choroidal structures and did not include assessment of anterior segment structures (such as corneal curvature, anterior chamber depth, and lens thickness).

Based on the findings and limitations of this study, future research could proceed in the following directions: (1) Prospective longitudinal studies should be conducted to dynamically monitor changes in retinal microcirculation and refractive status in children with ROP, thereby elucidating their potential causal relationship. (2) Larger sample sizes and longer follow-up periods are needed to assess the long-term effects of microcirculatory changes on refractive development during adolescence and adulthood. (3) Molecular biology techniques should be employed to elucidate the roles of VEGF and other angiogenic factors in retinal microcirculation remodeling and refractive development during the later stages of ROP. (4) Predictive models for refractive development should be constructed using OCTA parameters to facilitate early identification of high-risk children and implement individualized interventions.

In conclusion, this study confirms a significant correlation between macular retinal and choroidal structural characteristics and the current refractive status in children with ROP. An enlarged FAZ area, reduced SCP vessel density, and decreased choroidal thickness are closely associated with the development of myopia. Furthermore, serum VEGF levels are significantly correlated with macular microcirculatory changes, suggesting a potential role in regulating retinal microvascular remodeling in the later stages of ROP. The combined application of OCTA and serum VEGF measurement provides an objective basis for evaluating retinal microcirculatory changes and predicting refractive development in ROP, offering substantial clinical value for long-term visual monitoring and individualized intervention in preterm children.

## Data Availability

The original contributions presented in the study are included in the article/Supplementary Material, further inquiries can be directed to the corresponding author.
